# Metachronous osteoblastoma of the spine and osteoid osteoma of the femur

**DOI:** 10.1259/bjrcr.20150256

**Published:** 2015-08-13

**Authors:** Costantino Errani, Giancarlo Facchini, Giuseppe Rossi, Daniel Vanel, Alberto Righi, Ugo Albisinni, Davide Donati

**Affiliations:** ^1^ Orthopaedic Service, Rizzoli Institute, Bologna, Italy; ^2^ Radiology Service, Rizzoli Institute, Bologna, Italy; ^3^ Angiographic and Interventional Radiology Service, Rizzoli Institute, Bologna, Italy; ^4^ Pathology Service, Rizzoli Institute, Bologna, Italy

## Abstract

Osteoblastoma (OBL) and osteoid osteoma (OO) are usually solitary tumours, only rarely being multicentric. Herein, we report an unusual case in which a typical OBL of the spine was followed by an OO of the femur after a disease-free interval of 5 years. We believe that our unique case represents the first report of a metachronous OBL and an OO, and this presentation may confirm the close correlation between these two rare entities.

## Summary

Osteoblastoma (OBL) and osteoid osteoma (OO) are benign bone tumours with a very close histological resemblance. OBLs and OOs are usually solitary lesions, and are only rarely multicentric.^1^ To our knowledge, this is the first case report of a patient with a typical OBL of the spine followed by a typical OO of the femur after a disease-free interval of 5 years.

## Case report

**Figure 1.**
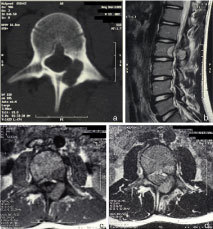
CT image shows (a) a well-limited lytic lesion of the posterior elements of L2 with sclerosis that indicates an inflammatory reaction in the vertebral body. MRI shows the peritumoral oedema, (b) sagittal *T*_2_ weighted, (c) axial *T*_1_ weighted and (d) *T*_2_ weighted images, with hypointense signal on *T*_1_ weighted images and hyperintense signal on *T*_2_ weighted images.

A 20-year-old male presented with knee pain that increased in intensity at night. The patient had a history of an OBL of the posterior spinal column treated 5 years ago. The OBL was diagnosed through a CT scan and an MRI that demonstrated an osteolytic lesion of the second lumbar vertebrae. The CT scan revealed a 2-–3 cm ovoid lesion in the posterior arch of the second lumbar vertebrae and an MRI of the lumbar spine confirmed a reactive, hypointense soft-tissue swelling in *T*_1_ with a mildly increased *T*_2_ signal (Figure 1). The patient was treated with curettage and the histological diagnosis was OBL.

**Figure 2.**
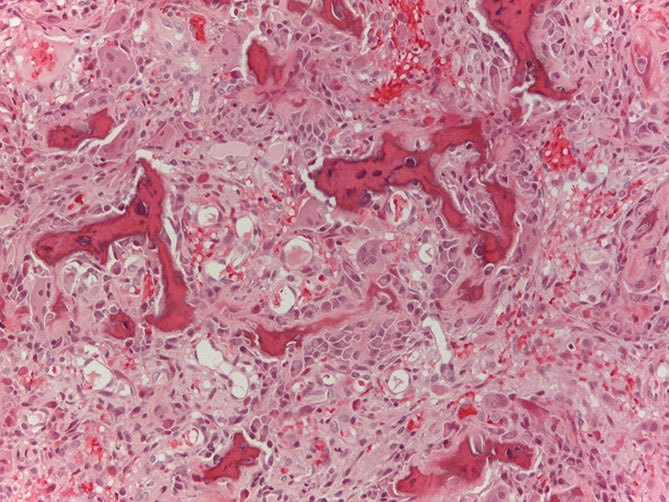
Microscopic examination shows an osteoblastoma: irregular woven bone, ectatic blood vessels, osteoblasts and giant cells are evident (haematoxylin and eosin, magnification 250×).

**Figure 3.**
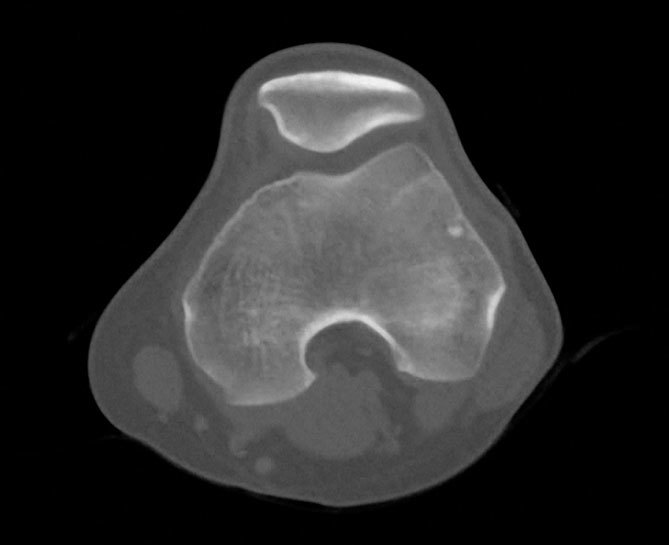
CT image of the knee shows the osteoid osteoma with a very small and calcified nidus.

**Figure 4.**
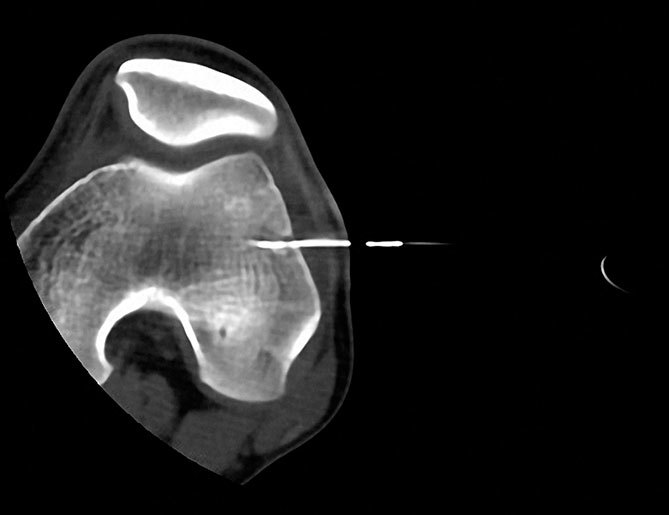
The final position of the radiofrequency electrode is shown, with the active tip inside the nidus.

**Figure 5.**
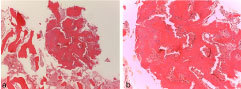
The sample removed with the biopsy and examined under a microscope shows an osteoid osteoma. The haematoxylin and eosin staining demonstrates a very sharp circumscription of the nidus (a), magnification 100×. Trabeculae are thin and form an inter-anastomosing network with abundant cement lines and a single layer of osteoblasts. The intertrabecular space is filled with fibrovascular stroma (b), magnification 200×.

At the time of our examination, we reviewed the histological slides of the previous diagnosis and the diagnosis of an OBL was confirmed (Figure 2). The patient presented with knee pain identical in character to the pain associated with the previous OBL of the spine. The knee pain was almost constant and had the typical tendency to increase in intensity during the night; it was relieved by non-steroidal anti-inflammatory drugs. There was no limitation of movement of the knee and no neurovascular deficit. The CT scan showed a small nidus without sclerosis and the MRI confirmed the nidus with an inflammation of the surrounding tissue (Figure 3). The bone scintigraphy, performed to rule out more lesions, showed increased activity in the left distal femur. Before starting radiofrequency ablation (RFA) treatment, a biopsy sample was obtained using the Bonopty set (Radi MS, Uppsala, Sweden). With the patient under spinal anesthesia, a core-needle biopsy was performed under CT guidance. Then, we placed the needle electrode inside the nidus (monopolar, non-refrigerated; SMK, Radionics, Burlington, MA), and RFA was performed through the same tract with a 5-mm radiofrequency probe heated to 90 °C for 5 min, using the radiofrequency generator (RFG-3C; Radionics) (Figure 4). The diagnosis was an OO (Figure 5). After a few days, the patient was completely asymptomatic and at the time of the most recent follow-up (1 year after treatment), the patient remained symptom free.

## Discussion

We herein report a unique case in which a typical OBL of a lumbar vertebra was followed by a typical OO of the femur. OBLs and OOs are members of the same family of benign bone-forming tumours. As the histological features may be identical, the lesion size is often used as a parameter for differential diagnosis (with lesions smaller than 1.5–2 cm being classified as OOs and larger lesions as OBLs).^2,3^ Whereas patients with OO are usually treated with RFA, those with OBL are usually treated with local resection.

Although both OBL and OO are most often monostotic, they may rarely occur in more than one bone, either synchronously or metachronously.^1^ In a review of the English literature, there are only eight patients with multifocal OO: three had synchronous and five had metachronous lesions.^1,4^ Even rarer still is multifocal OBL; only three cases have been published.^1,5,6^

In the case of our patient, the tumours were anatomically distant and were separated in time by a 5-year interval. It is possible that this occurrence represents nothing more than a statistical coincidence. However, although the aetiology of OOs and OBLs remains unknown, it is possible that a metachronous presentation of these rare tumours may be a manifestation representing a genetic correlation between them. We believe that our case report is important not only to confirm the possibility of a metachronous presentation of mesenchymal tumours, but also to underline a possible pathogenetic correlation between an OO and an OBL as part of the same family of tumours producing osteoid and woven bone.

The genetic profile of an OO and an OBL has not yet been established but there are a few reports showing genetic aberrations of both tumours.^7–10^ Although a plausible genetic link between OOs and OBLs is not apparent, the possibility should be investigated starting with their morphological similarity. In fact, at the cellular level, an OBL is identical to an OO; both tumours show rich vascularization, an irregular osteoid with osteoblasts and often osteoclast-type multinucleated giant cells.^9^ The loss or rearrangement of the long arm of chromosome 22 was found in both OBLs and OOs.^7,9^ Baruffi et al^7^ showed the same alteration involving the long arm of chromosome 22 in two cases of OOs. They suggested that this chromosomal alteration may affect critical genes involved in the regulation of cell proliferation.^7^ The available data also indicate that a candidate target gene for OBL development may reside in the long arm of chromosome 22.^9^ The pathogenetic importance of these aberrations is not known but the possible deleted genes seem to be involved in osteogenesis and tumorigenesis.^9^

We can speculate that the data provides two different possibilities: either the patient has two different tumours that are histologically identical and similar in their genetic profile or the OBL and OO have the same pathology, suggesting that the differential diagnosis based on size is weak. Furthermore, similar to other benign mesenchymal tumours, their metachronous presentation may represent a multifocal involvement of the same disease. In fact, the possible existence of benign metastasis is supported by the behaviour of giant cell tumours and epithelioid haemangioma.^11^

Our case report shows that a metachronous presentation of OBLs and OOs is an exceptional event, but must be considered in cases of patients with a history of previous OBL or OO who present with a deep and continuous pain in a different site, increasing in intensity during the night and relieved by oral consumption of non-steroidal anti-inflammatory drugs. The genetic mechanisms underlying OBL and OO development are largely unknown and no obvious genetic correlation between these two rare entities has been identified. However, we believe that the case of our patient represents the first report of metachronous OBL and OO, and this presentation may confirm the close correlation between these two rare entities.

## Learning Points

1. A metachronous presentation of OBLs and OOs is an exceptional event, but must be considered in cases of patients with a history of previous OBL or OO who present with a deep and continuous pain in a different site, increasing in intensity during the night and relieved by oral consumption of non-steroidal anti-inflammatory drugs.
2. A metachronous presentation of OBLs and OOs may confirm the close correlation between these two rare entities and underlines a possible pathogenetic correlation between an OO and an OBL as part of the same family of tumours producing osteoid and woven bone.
3. It is also possible that a metachronous presentation of OBL and OO may be a manifestation representing a genetic correlation between them.
